# Evaluation of relaxant responses properties of cinnamon essential oil and its major component, cinnamaldehyde on human and rat corpus cavernosum

**DOI:** 10.1590/S1677-5538.IBJU.2019.0016

**Published:** 2019-01-29

**Authors:** Alev Onder, Didem Yilmaz-Oral, Igor Jerkovic, Alp Ozgur Akdemir, Serap Gur

**Affiliations:** 1 Department of Pharmacognosy Faculty of Pharmacy Ankara University Ankara Turkey Department of Pharmacognosy, Faculty of Pharmacy, Ankara University, Ankara, Turkey;; 2 Department of Pharmacology Faculty of Pharmacy Ankara University Ankara Turkey Department of Pharmacology, Faculty of Pharmacy, Ankara University, Ankara, Turkey;; 3 Department of Pharmacology Faculty of Pharmacy Cukurova University Adana Turkey Department of Pharmacology, Faculty of Pharmacy, Cukurova University, Adana, Turkey;; 4 Department of Organic Chemistry Faculty of Chemistry and Technology University of Split Split Croatia Department of Organic Chemistry, Faculty of Chemistry and Technology, University of Split, Split, Croatia;; 5 Department of Urology Ankara Numune Education and Research Hospital Ankara Turkey Department of Urology, Ankara Numune Education and Research Hospital, Ankara, Turkey

**Keywords:** cinnamic aldehyde [Supplementary Concept], Penile Induration, Humans

## Abstract

*Cinnamomum cassia* (Cinnamon) is a well-known traditional medicine with therapeutic benefits for centuries. We evaluated the effects of cinnamon essential oil (CEO) and its main component cinnamaldehyde (CA) on human corpus cavernosum (HCC) and rat CC. The essential oil of cinnamon was analyzed for the confirmation of the oil profile. HCC specimens from patients undergoing penile prosthesis surgery (age 48-69 years) were utilized for functional studies. In addition, erectile responses in anesthetized control and diabetic rats were evaluated in vivo after intracavernosal injection of CEO and CA, and rat CC strips were placed in organ baths. After precontraction with phenylephrine (10µM), relaxant responses to CEO and CA were investigated. CA (96.9%) was found as the major component. The maximum relaxation responses to CEO and CA were 96.4±3.5% and 96.0±5.0% in HCC and 97.5±5.5% and 96.8±4.8% in rat CC, respectively. There was no difference between control and diabetic rats in relaxation responses to CEO and CA. The relaxant responses obtained with essential oil and CA were not attenuated in the presence of nitric oxide synthase (NOS) inhibitor, and soluble guanylate cyclase inhibitor (sGS) in CC. In vivo, erectile responses in diabetic rats were lower than in control rats, which was restored after intracavernosal injection of CEO and CA. CEO and CA improved erectile function and relaxation of isolated strips of rat CC and HCC by a NO/cGMP-independent mechanism. Further investigations are warranted to fully elucidate the restorative effects of CEO and CA on diabetic erectile dysfunction.

## INTRODUCTION

Erectile dysfunction (ED) is one of the most common health problems for men. In the current treatment for ED, phosphodiesterase-5 inhibitors (PDE-5i) are considered the first-line therapy (1). Plant-derived products have been used for the management of ED for a long time, and over 15% of men use natural-based therapies (2). Yohimbine, Korean ginseng, and ginkgo are popular examples used as male sexual performance enhancers in traditional medicine (3). Previous clinical trials with Yohimbine demonstrated that the combination with L-arginine was effective in improving erectile function in patients with mild to moderate ED (4). In addition, oral gavage with Ginsenoside Rg3 (100mg/kg) normalized in vivo erectile responses in a diabetic rat model (5). Furthermore, a clinical trial showed the positive effects of ginseng on IIEF-5 scores compared with placebo. The treatment with ginseng was effective in four of the five IIEF-15 domains (2). Another study indicated that four weeks of daily treatment with high-dose Ginkgo biloba extract significantly increased erectile function in comparison with the vehicle-only treatment (6). Recently, we found that the treatment with pomegranate juice improved ED in a diabetic rat model (7).

The genus *Cinnamomum* (Cinnamon) Schaeff (Lauraceae) comprises over 250 species, which are aromatic evergreen shrubs and trees growing in tropical rain-forests in a majority of Asia (8). Cinnamon is one of the oldest spices, which has been used in many cultures for centuries. *Cinnamomum cassia* or Chinese cinnamon is one of the most important and popular species in this genus. The bark and stem of the plant are an excellent natural source of aromatic spices (8). *Cinnamomum cassia* includes several secondary metabolites such as coumarins, diterpenoids, polyphenols, and essential oils. The essential oil of the plant bark mainly contains cinnamaldehyde (CA) besides cinnamic acid (9). Moreover, the plant is well-known for its medicinal properties for the treatment of some diseases with many critical pharmacological effects such as platelet anti-aggregation, antidiabetic, anti-inflammatory and antioxidant (9-12).

In traditional oriental herbal medicine, *Cinnamomum cassia* extract (8) has been suggested to improve sexual performance (13). Moreover, the methanolic extract of *Cinnamomum cassia* improved the sexual function in aged rats by increase in smooth muscle/collagen ratio and decrease in oxidative stress in rat penile smooth muscle (10). Furthermore, the methanol extract of the plant barks has been potentially inhibited arginase activity on isolated rat corpus cavernosum (CC) smooth muscles (14).

In the present study, the cinnamon essential oil (CEO, from *Cinnamomum cassia* Blume) was evaluated for the potential effect on i*n vitro* on isolated human CC (HCC), diabetic and control rat CC, as well as *in vivo* erectile function in diabetic rats. In addition, the essential oil of *Cinnamomum cassia* Blume was analyzed by GC (Gas Chromatography) and spectroscopy (GC/MS) for the identification and confirmation of the major components. To understand the effect of related compounds in diabetic conditions, its major compound CA-induced relaxation responses were evaluated on both *in vitro* and *in vivo* in diabetic rats compared with control rats.

## MATERIALS AND METHODS

### Plant material

The essential oil of *Cinnamomum cassia* Blume has been supplied from Heal with Essential Oil Company (Florida, USA).

### GC/Flame Ionization Detector (FID) and GC/MS analysis procedure for the identification of essential oil components.

Analyses of CEO (diluted 1:100 v/v with n-pentane: diethyl ether 1:2 v/v) were performed using GC/FID and GC/MS in triplicate. GC analyses were carried out on a gas chromatograph (Agilent Technologies, CA, USA) equipped with FID. Chromatographic separations were performed on a 30m x 0.25mm HP-5MS capillary column [(5%-phenyl)-methylpolysiloxane, Agilent J and W GC column] with a coating thickness of 0.25μm. The oven was temperature-programmed isothermal at 70°C for 2 min and then increased to 200°C at a rate of 3°C/min and held isothermal for 15 min. Helium at 1mL/min was used as a carrier gas. The injector temperature was 250°C, and the detector temperature was 300°C. The injected volume was 1μL, and the split ratio was 1:50. The mass detector operated in the electron impact ionization mode at 70 eV; the mass range was m/z 30-300, and the ion source temperature was 280°C. The volatile compound separation was obtained using the same column and oven temperature program as previously described. The individual peaks were identified by the comparison of their retention indices (relative to C9-C25n-alkanes for HP-5MS column), as well as by comparing their mass spectra with the Wiley 275MS library (Wiley, New York, USA) and NIST98 (Gaithersburg, Germany) mass spectral database. The percentage composition of the samples was computed from the GC peak areas using the normalization method (without correction factors).

### HCC tissue strips

A total of 16 men with ED and/or Peyronie’s disease were enrolled in the present study with consent under Institutional Review Board guidelines. CC samples were obtained from the patients (age: 48-69) who had undergone penile prosthesis surgery. HCC tissue strips were placed in cold Krebs isotonic solution [consisting in (mM): NaCl, 118; NaHCO_3_, 25; glucose, 5.6; KCl, 4.7; KH_2_PO_4_, 1.2; MgSO_4_ 7 (H_2_O), 1.17; and CaCl_2_ 2H_2_O, 2.5] and immediately transported (between 15-30 min) to the laboratory for in vitro experiments.

### Animal experiments

Ten adult male Sprague-Dawley rats (300-350g) were randomly divided into control and diabetic groups. Diabetic rats received a single intraperitoneal injection of streptozotocin (40mg/kg), which was dissolved in a citrate buffer (pH=5.5). Measurement of blood glucose levels was performed using an Accu-Chek glucometer (Roche Diagnostics, Indianapolis, IN) after the induction of diabetes. Rats were housed in separate cages and were provided with food and water ad libitum in a temperature-controlled room (22±1°C) that was artificially lit from 7:00 AM to 7:00 PM daily. The present study was approved by the Institutional Animal Care and Use Committee of Ankara University (2014-9-66).

### In vivo studies

Eight weeks after the induction of diabetes, rats were anesthetized with sodium pentobarbital (50mg/kg, i.p.) to measure intracavernosal pressure (ICP). The trachea was cannulated using polyethylene-240 tubing to maintain the constant airway. The carotid artery was also cannulated (polyethylene-50 tubing) to measure mean arterial pressure (MAP) using a transducer (Statham, Oxnard, CA) attached to a data acquisition system (Biopac MP 100 System, Santa Barbara, CA). A 25-gauge needle filled with 250U/mL of heparin and connected to the polyethylene tubing was inserted into the right crura of the penis and connected to the pressure transducer to measure ICP continuously. The right major pelvic ganglion and cavernous nerve (CN) were identified. A stainless steel bipolar hook electrode was placed around the CN posterolateral to the prostate for the stimulation. The CN was stimulated (2.5, 5 and 7.5 V, 15 Hz, 30-second pulse width) with a square pulse stimulator (Grass Instruments, Quincy, MA), and MAP and ICP were continuously measured. The measurements were repeated after the intracavernosal administration of CEO and CA (1µM) in the diabetic group.

### Measurement of isometric tension in CC strips

CC was placed in a petri dish containing Krebs-bicarbonate and was oxygenated with a mixture of 95% O_2,_ and 5% CO_2_. On average 4 strips of HCC tissue (1 x 1 x 8mm) were prepared from each cavernosal sample. Strips were suspended in 20mL organ bath chambers (Radnoti Glass Technology Inc, Monrovia, California) with one end fixed to a tissue holder and the other secured to a force transducer (FT03 Grass Instruments, Quincy, Massachusetts). Organ chamber temperature was maintained at 37°C via a circulating water bath. After the placement of tissue strips in the organ bath chamber, the preparations were allowed to equilibrate for about 90 min, and the bath solution was replaced every 15 min. The CC strips were pre-contracted with phenylephrine (PE, 10µM) and allowed to relax in response to the administration of CEO (26, 52 and 104mg) and CA (26, 52 and 104mg). The relaxant responses to CEO and CA were recorded before and after the administration of the nonspecific NO synthase (NOS) inhibitor, L-NAME (NG-nitro-L-arginine methyl ester, 100µM) and soluble guanylyl cyclase (sGC) inhibitor, ODQ (1H-[1,2,4] oxadiazole [4,3-a] quinoxaline-1-one, 30µM). CC strips were incubated with inhibitors for 20 min before obtaining the relaxation curves.

Sodium nitroprusside-(SNP, 10nM) and sildenafil (10nM)-induced relaxation responses were evoked after precontraction of CC strips with PE (10^5^ M) in the presence or the absence of CEO (26mg) in HCC.

### Drugs and chemicals

All drugs, as well as CA, were purchased from Sigma-Aldrich Chemical Company (St. Louis, MO, USA).

### Data analysis

All values are expressed as mean±SEM. Statistical differences were determined by one-way analysis of variance (ANOVA) with repeated measures followed by Bonferroni post-test performed using Prism 4 statistical analysis packages for Windows (GraphPad Software, La Jolla, CA, USA). A p-value <0.05 was considered significant.

## RESULTS

### Identification of the essential oil components

In essential oil analysis, components of CEO (99.3%) were identified. CA (96.9%) has been determined as a major component in the oil of cinnamon as seen in Table-1.

**Table 1:** The essential oil composition of *Cinnamomum cassia* Blume.

Peak number	Compounds	RRI	Area %
1	Benzaldehyde	965	0.3
2	Phenylacetaldehyde	1048	0.2
3	*trans*-Cinnamic aldehyde	1277	96.9
4	trans-Cinnamic acid	1457	1.9

### CEO and its major compound CA-induced relaxation of HCC strips and involvement of NO/sGC pathway.

**Figure 1:**
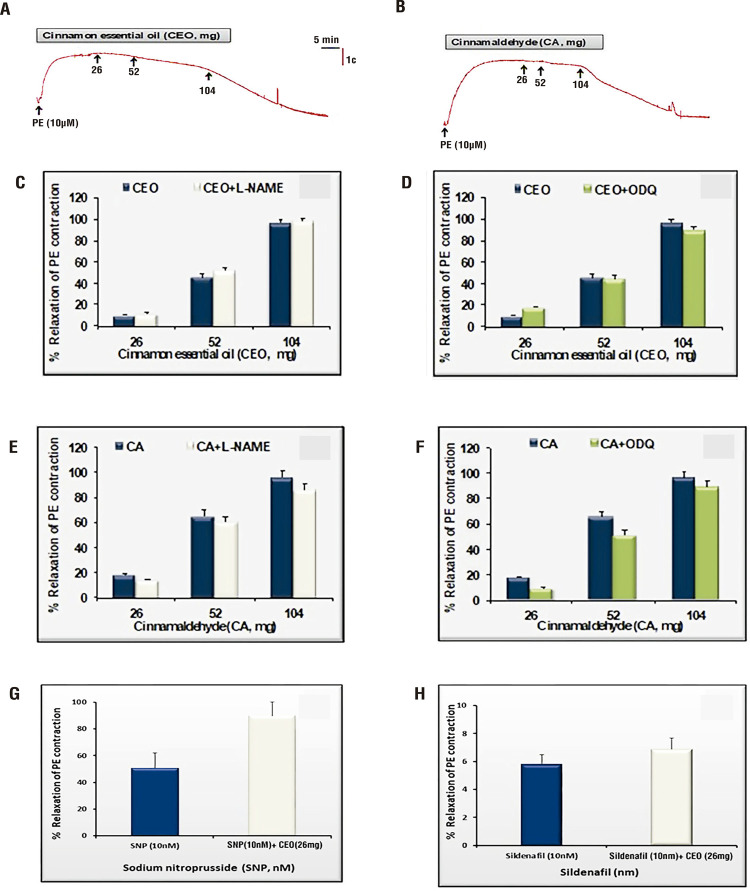
Representative traces are showing the relaxation effect of cinnamon essential oil (CEO, A) and cinnamaldehyde (CA, B) on phenylephrine (PE) pre-contracted human corpus cavernosum (HCC) strips. Relaxation-response curves for CEO and CA in HCC strips after pre-contraction with PE (10µM) in the presence of L-NAME (100µM, n=8), ODQ (30µM, n=7). Relaxation-response curves for sodium nitroprusside (SNP, G) and sildenafil (H) in HCC strips after pre-contraction with PE (10µM) in the presence of CEO (26mg, n=8). Data represent the mean SEM of 6-8 observations.

Representative relaxation traces of CEO (Figure-1A) and its major compound CA (Figure-1B) in isolated HCC are shown in Figure-1. The maximum relaxation induced by CEO was 96.4±3.5%, was not inhibited in the presence of either L-NAME (98±4%) or ODQ (90±3%) (Figures 1C and D). Also, CA-induced maximal relaxation response was 96±5%. Similarly, the maximal relaxation response to CA did not change in the presence of L-NAME (86.0±4.5%) and ODQ (89±4%) (Figures 1E and F).

### Effect of CEO on SNP-and sildenafil-mediated relaxation of HCC

SNP-induced relaxation response at 10nM dose was increased in the presence of CEO (26mg), which was not statistically significant (Figure-1G). There was no difference in sildenafil-induced relaxation response at 10nM between the absence and presence of CEO (26mg, Figure-1H).

### *In vitro* responses of rat CC strips

**Figure 2:**
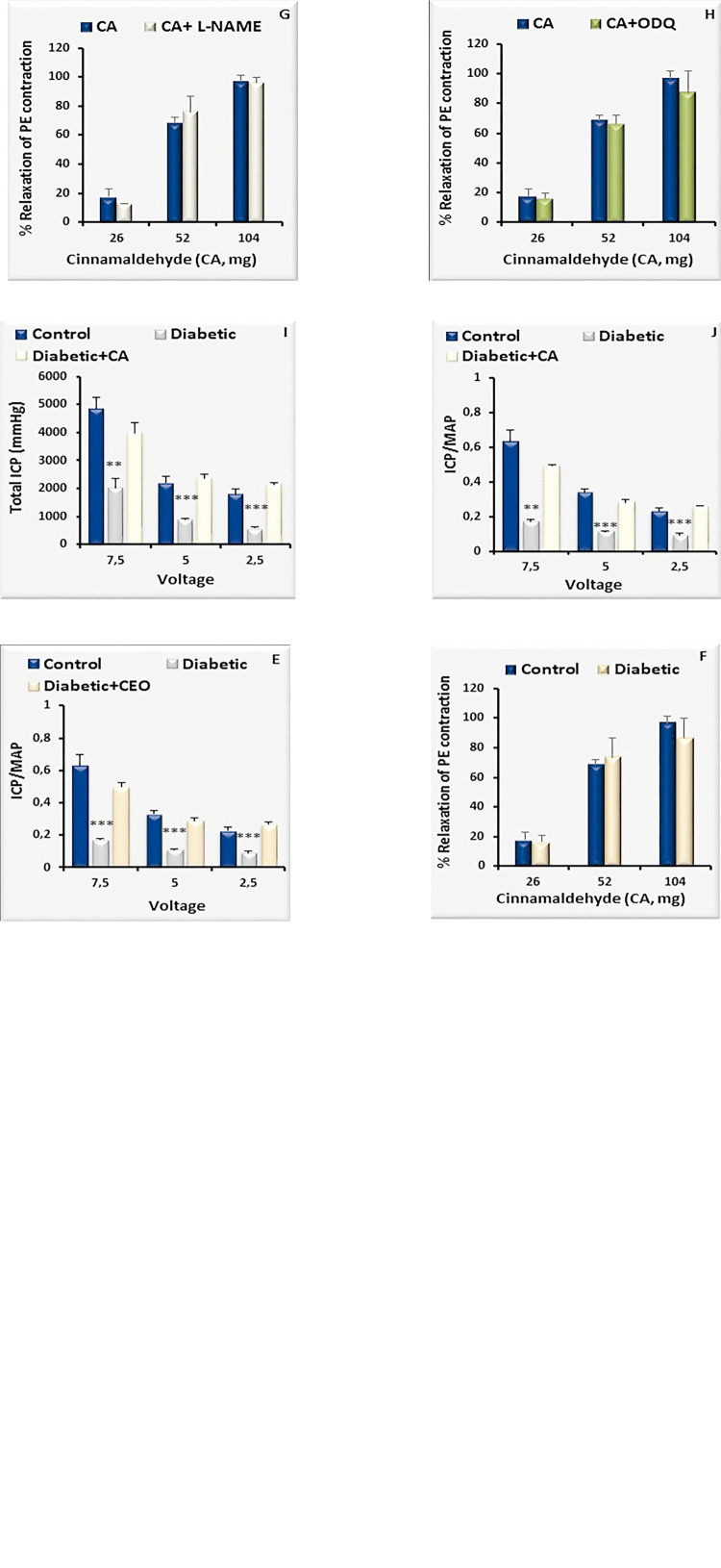
Relaxation-response curves for cinnamon essential oil (CEO) and cinnamaldehyde (CA) in rat corpus cavernosum (CC) strips after pre-contraction with phenylephrine (PE, 10µM) in the presence of L-NAME (100µM, n=8), ODQ (30µM, n=7). In vivo intracavernosal effect of CEO and CA on the penile erection of diabetic and control rats. Data represent the mean±standard error of the mean of 6-8 observations.

The maximal relaxation induced by CEO was 97.5±5.5%, which was not altered in diabetic rats (87.3±1.0, Figure-2A). The relaxant responses to CEO were not inhibited by the presence of either L-NAME (95±6%) or ODQ (90±2%) (Figure 2B and C). Moreover, CA-induced maximal relaxation response in control and diabetic rats were 96.8±4.8% and 86.5±13.6%, respectively (Figure-2F). The maximal relaxation response to CA did not change in the presence of L-NAME or ODQ (Figures 2G and H).

**P <01, ***P <001 vs. control value.

### In vivo erectile responses in both groups

Total ICP and ICP/MAP values for erectile responses in diabetic rats were lower than those in control rats (P <01; Figure-2). After the intracavernosal administration of CEO (Figures 2 D and E) and CA (Figures 2I and J), the total ICP and the ICP/MAP values were restored in the diabetic rats at all voltage levels (Figure 2).

## DISCUSSION

This is the first report that provides basic mechanistic information concerning CEO and CA-induced dose-dependent relaxation in human and rat CC. The major findings of the present study show that (1) CEO and CA relax human and rat CC in a concentration-dependent manner; (2) NO-cyclic guanosine monophosphate (cGMP) pathway is not involved in the relaxation response to CEO and CA; and (3) CEO and CA improve in vivo erectile function in diabetic rats.

In the current study, CA (96.9%) has been determined as a major component in the oil of cinnamon by GC and GC/MS analyzes. In previous studies, CA has also been found as a major component of 90.22% in *Cinnamomum cassia* barks (15); 90% in Vietnamese Cinnamomum cassia barks (16).

The present study showed that CA and CEO remarkably induced relaxation of HCC and rat CC strips. There are no previous reports on the effects of CEO and CA on human and rat CC. However, Alotaibi (17) recently demonstrated that cinnamon extract decreased the force of uterine contraction, even when the uterus was stimulated by agonists. The maximum relaxation induced by CEO was not inhibited in the presence of either L-NAME or ODQ. Similarly, the maximal relaxation response to CA did not change in the presence of L-NAME and ODQ. These results suggest that the relaxation effect of CEO and CA is not mediated through the NO/cGMP pathway. There are few studies including these compounds and interactions with the NO/cGMP system. Davaatseren et al. (18) recently reported that trans-cinnamaldehyde, an active compound of cinnamon did not affect the production of NO. In addition, the relaxation response to CA in rat aorta and porcine coronary artery did not alter in the presence of NOS and sGC inhibitors (19, 20). Furthermore, the methanol extract of *Cinnamomum zeylanicum* stem bark displayed antihypertensive and organ protective effects in L-NAME-induced hypertensive rats (21).

In the present study, the SNP-induced relaxation response was enhanced after incubation with CEO, which was not statistically significant. In addition, there was no difference in sildenafil-induced relaxation response between the absence and presence of CEO. The previous study showed that endothelium-independent relaxation in mice aorta did not change after the incubation with CA (22). To our knowledge, this is the first data regarding the effects of CEO on cavernosal smooth muscle.

We found that ICP/MAP and total ICP values for erectile responses in diabetic rats were lower than those in control rats. After the intracavernosal administration of CEO and CA, the ICP/MAP and the total ICP values were restored in diabetic rats at all voltage levels. Furthermore, CA and CEO remarkably relaxed both control and diabetic rat CC. We suggest that these compounds may have a restorative effect involving in hyperglycemia-induced reactive oxygen species (ROS) production. In a study by Wang et al. (22), CA is a crucial flavor compound in CEO that enhanced the antioxidant defense against ROS by activating the transcription factor Nuclear factor (erythroid-derived 2)-like 2 (NF-E2-related factor 2) indicating a cardiovascular protective effect. Based on these findings, CA preserved endothelial function under high glucose conditions, but the underlying mechanism is unknown. In addition, Raffai et al. (20) showed that CA-loaded and poly-CA micelles include vasodilator properties, and thus may be used both to relieve coronary vasospasm and for therapeutic drug delivery. Lee et al. suggested that the hypoglycemic activity and pancreas-protective effects of cinnamon in diabetic rats induced by streptozotocin were observed (11). Li et al. (12) demonstrated that cinnamon polyphenols could exert the hypoglycemic and hypolipidemic effects through improving its anti-oxidative capacity, as well as attenuating cytotoxicity via inhibition of inducible NOS, nuclear factor kappa B activation in diabetic mice. We suggest that these compounds could have restorative effects involving in hyperglycemia-induced ROS production under diabetic conditions.

Cinnamon is a dietary component that has been demonstrated to include biologically active substances that regulate blood glucose by insulin-mimetic properties (23). Several clinical trials exhibited that Cinnamon and its extracts achieved a therapeutic effect on diabetic patients (24, 25). In clinical trials, cinnamon displayed positive effects on glycemic control lipid markers in type 2 diabetes populations (24, 26). In light of the previous data supporting cinnamon dietary supplement improves glycemic parameters. Thus, a cinnamon dietary supplement may be beneficial in deterring the incidence of diabetes-induced ED by preventing glycemic parameters. It remains unknown that the benefits of different cinnamon extract supplementations on the prevention and treatment of diabetes-induced ED. Furthermore, these results may be supported further clinical and preclinical studies using combinations of cinnamon dietary supplement and PDE5i for the treatment of diabetic ED, especially in patients who do not respond to PDE5i therapy.

Overall, our results suggest that cinnamon and its major compound, CA may be effective, although a number of some limitations that should be discussed to help shape future research. For instance, limited trial numbers, total sample sizes, methodological differences, imprecise standardization of herbal extracts used, and unclear risk of bias may decrease interest for possible utility in clinical practice. Several compounds have shown promise in vitro and animal studies, but do not provide any clinical benefit. Previous data obtained from in vitro cell cultures and in vivo animal experiments must be translated into human activities. In addition, more rigorous clinical trials in the field are required before the consumption of herbal dietary supplements can be definitively suggested for the treatment of ED.

In conclusion, the present study demonstrates that CEO and CA induce relaxation in HCC and rat CC in a NO/cGMP-independent manner. Our investigation revealed novel biological functions of CEO and CA for erectile function. Further research is required to address the underlying molecular mechanisms of CEO and CA responsible for cavernosal smooth muscle relaxation.
